# Revisiting the management of term breech presentation: a proposal for overcoming some of the controversies

**DOI:** 10.1186/s12884-020-2831-4

**Published:** 2020-05-03

**Authors:** Lionel Carbillon, Amelie Benbara, Ahmed Tigaizin, Rouba Murtada, Marion Fermaut, Fatma Belmaghni, Alexandre Bricou, Jeremy Boujenah

**Affiliations:** 1 Department of Obstetrics and Gynecology, Sorbonne Paris Nord University, Assistance Publique – Hopitaux de Paris, Avenue du 14 juillet, Hôpital Jean Verdier, 93140 Bondy Cedex, France; 2 Department of Obstetrics and Gynecology, Assistance Publique – Hôpitaux de Paris, Hôpital Jean Verdier, Bondy, France

**Keywords:** Term breech delivery, Small-for-gestational-age, Foetal growth restriction, Oligohydramnios, Delivery route, Pelvimetry, Perinatal mortality, Perinatal morbidity, Severe maternal morbidity

## Abstract

**Background:**

The debate surrounding the management of term breech presentation has excessively focused on the mode of delivery. Indeed, a steady decline in the rate of vaginal breech delivery has been observed over the last three decades, and the soundness of the vaginal route was seriously challenged at the beginning of the 2000s. However, associations between adverse perinatal outcomes and antenatal risk factors have been observed in foetuses that remain in the breech presentation in late gestation, confirming older data and raising the question of the role of these antenatal risk factors in adverse perinatal outcomes. Thus, aspects beyond the mode of delivery must be considered regarding the awareness and adequate management of such situations in term breech pregnancies.

**Main body:**

In the context of the most recent meta-analysis and with the publication of large-scale epidemiologic studies from medical birth registries in countries that have not abruptly altered their criteria for individual decision-making regarding the breech delivery mode, the currently available data provide essential clues to understanding the underlying maternal-foetal conditions beyond the delivery mode that play a role in perinatal outcomes, such as foetal growth restriction and gestational diabetes mellitus. In view of such data, an accurate evaluation of these underlying conditions is necessary in cases of persistent term breech presentation. Timely breech detection, estimated foetal weight/growth curves and foetal/maternal well-being should be considered along with these possible antenatal risk factors; a thorough analysis of foetal presentation and an evaluation of the possible benefit of external cephalic version and pelvic adequacy in each specific situation of persistent breech presentation should be performed.

**Conclusion:**

The adequate management of term breech pregnancies requires screening and the efficient identification of breech presentation at 36 weeks of gestation, followed by thorough evaluations of foetal weight, growth and mobility, while obstetric history, antenatal gestational disorders and pelvis size/conformation are considered. The management plan, including external cephalic version and follow-up based on the maternal/foetal condition and potentially associated disorders, should be organized on a case-by-case basis by a skilled team after the woman is informed and helped to make a reasoned decision regarding delivery route.

## Background

The ideal management of women with term breech presentation remains a matter of intense debate. The rate of vaginal delivery has steadily declined in the last decades of the last century [1]. In 2000, the Term Breech Trial (TBT) Collaborative Group concluded that a composite variable combining perinatal and neonatal mortality or serious neonatal morbidity was significantly lower in the planned caesarean section (CS) group than in the planned vaginal birth group [2], which marked an apparent turning point in this controversy. Based on the short-term outcomes presented in the TBT study, the Royal College of Obstetricians and Gynaecologists (RCOG) [3] and the American College of Obstetricians and Gynecologists (ACOG) [4] recommended over the next few years that all women with persistent singleton breech presentation at term should undergo a planned CS delivery. An important and almost immediate impact on the practice was also observed in some countries that previously had a high proportion of vaginal breech deliveries [5]. TBT was the largest randomized trial ever published on the term breech mode of delivery. However, despite its undeniable strengths, a number of weaknesses have been identified. Specifically, there was a lack of adherence to strict criteria for vaginal birth in an important proportion of the included patients and nonoptimal methods of labour management as recognized by the TBT group itself [6–8]. In addition, when the TBT Collaborative Group published the 2-year analysis of paediatric outcomes, despite a large (greater than 50%) post-randomization loss to follow-up [9], these researchers found no reduction in the risk of death or neurodevelopmental delay in children at 2 years of age, thus raising questions regarding the real lessons to be drawn from this trial. Using multiple logistic regression analyses, the TBT group also reported [10] that the risk of maternal morbidity was lowest following vaginal birth (odds ratio [OR] 1.0) and highest following CS after active labour (36.1% in the TBT) (OR 3.33; 95% CI 1.75–6.33, *P* < 0.001), particularly after a short second stage < 30 min (OR 0.25; 95% CI 0.11–0.57, P < 0.001) [9].

Later, population-based retrospective studies helped refine the consequences of applying recommendations of systematically planned CS for women with term breech presentation at the population level. Hartnack Tharin et al. [11] found that the rate of CS for term breech deliveries increased from 79.6 to 94.2% between 1997 and 2008 in Denmark, while intrapartum or early neonatal mortality decreased from 0.13 to 0.05% [relative risk (RR) 0.38 (95% CI 0.15–0.98)], which was a significant but lower reduction than the difference reported in the TBT. Using the Dutch National Perinatal Registry from 1999 to 2007, Vlemmix et al. [12] stated that after publication of the TBT, the elective CS rate increased from 24 to 60%, and overall perinatal mortality and short-term morbidity decreased. In contrast, these outcomes remained stable in the planned vaginal birth group. However, the authors estimated that 338 CS deliveries would need to be performed to prevent one perinatal death, and Schutte et al. [13] estimated the perinatal case fatality rate for elective CS for breech presentation in 2000–2002 at 0.47/1000 operations. At the same time, in the Netherlands the incidence of severe maternal morbidity (SMM) was estimated at 6.4/1000 during an elective CS compared with 3.9/1000 during an attempted vaginal delivery (OR 1.7; 95% CI 1.4–2.0), with an increased risk for SMM in the next pregnancy (OR 3.0; 95% CI 2.7–3.3) [14], despite the numerous facilities and adequate resources allocated to perinatal care in such a high-income country.

On the other hand, new guidelines were published in 2009 by the Society of Obstetricians and Gynaecologists of Canada (SOGC) stating that “planned vaginal delivery is reasonable in selected women with a term singleton breech foetus”. Afterwards, a study [15] including 52,671 breech deliveries in Canada (2003–2011) reported in 2011 that vaginal deliveries increased from 2.7% in 2003 to 3.9%. In this study, a concomitant increase in composite neonatal mortality and morbidity rates was observed with an adjusted rate ratio of 3.60 (95% CI 2.50–5.15), compared with CS without labour [15]. Moreover, CS with labour also increased from 8.7 to 9.8%, highlighting the particular difficulty in returning to previous practices after the clinical skills required to conduct a vaginal breech delivery have declined [15, 16].

Some authors recently considered that “the TBT recommendations should be withdrawn” [6], while others still consider that the “results (of the TBT) are generalizable” [16, 17]. Nevertheless, national guideline bodies have partially reversed their recommendations based on these discussions [18–20]. However, as rightly noted by Joseph et al. [16], the availability of clinical skills has declined in some of these countries, raising concerns from a pedagogic resident education and training standpoint [16]. In this regard, a meaningful role could be given to the possibility of training by simulation in building and maintaining specific skills and competencies for vaginal breech delivery.

A new meta-analysis [21] and several large-scale epidemiologic datasets from medical birth registries [22–24] recently evaluated risk factors associated with adverse perinatal outcomes in planned vaginal breech labours at term. These investigations were conducted in countries that have not abruptly modified their policies and that have continuously applied similar strict criteria over the last several decades for individual decision-making in cases of term breech presentation. We believe that the time has come to go beyond the sole question of delivery mode in the management of these situations.

## Discussion

### Term breech presentation: are we asking the right questions?

It now appears time to expand our thinking and, considering recent important data that help elucidate the underlying significance of persistent breech presentation at term, to offer more dynamic and multidisciplinary insight into the management of these cases.

Indeed, similar to some older studies [25–27], several recent population-based studies [22, 23] strongly suggest that the increased risk observed in foetuses that remain in the breech presentation at term is closely linked to antenatal or underlying disorders that may be associated with the breech presentation and is not solely due to the mode of delivery. Because adverse outcomes can be caused by underlying or gestational disorders, any discussion that is limited to delivery mode seems too restrictive and does not address the whole issue.

### Most recent large-scale data

#### Deterministic or accidental breech presentation?

In a recent Finnish population-based case-control study including all singleton deliveries from 1 January 2005 to 31 December 2014 and excluding preterm deliveries, antepartum-diagnosed stillbirths, placenta previa and infants with congenital malformations (499,206 foetuses at term), Macharey et al. [22] evaluated the antenatal risk factors associated with adverse perinatal outcomes in planned vaginal breech labour at term. They found that the stillbirth rate was significantly higher in cases of planned vaginal breech labour than in cases of cephalic presentation (0.2 vs 0.1%, respectively), which was correlated with foetal growth restriction, oligohydramnios, gestational diabetes mellitus (GDM) and a history of CS. Furthermore, in another recent survey based on the same cohort of mother-neonate dyads that also excluded congenital malformations, placenta previa and prelabour stillbirths [23], this same group showed that breech presentation at term was significantly associated with antenatal stillbirth and a number of individual obstetric risk factors for adverse perinatal outcomes, including oligohydramnios, foetal growth restriction, gestational diabetes, history of CS section and congenital anomalies. Among all planned singleton vaginal deliveries with the foetus in the breech presentation at term, a composite adverse perinatal outcome defined as umbilical arterial pH < 7.00, 5-min Apgar score below 7 and/or neonatal mortality during the first 6 days of life (excluding stillbirth) was associated with foetal growth restriction (aOR 2.94 [1.30–6.67]), oligohydramnios (adjusted OR 2.94 [1.15–7.81]), gestational diabetes (aOR 2.89[1.54–5.40]), and a history of CS (aOR 2.94 [1.28–6.77]).

In another recent population-based study based on perinatal data of all (650,968) children born in Norway from 1999 to 2009 [24], the authors recognized the limitations of most registry-based studies, as the selection of women with breech presentation and planned vaginal delivery was based on criteria that might have identified pregnancies with a lower risk of adverse outcomes compared with those selected for CS delivery. Moreover, in this study [24], the intrapartum conversion of some of the planned vaginal deliveries to an emergency CS delivery may have increased the risk for adverse outcome in the CS group. However, Bjellmo et al. [24] conducted an innovative analysis comparing breech deliveries to vaginal cephalic births. Thus, they showed that singleton children born at term without congenital malformations had a higher risk for stillbirth and neonatal mortality if they were born in the breech presentation “regardless of whether they were born vaginally or by CS delivery” (0.9 per 1000 in those actually delivered vaginally and 0.8 per 1000 in those actually born by CS delivery) compared with those born by vaginal cephalic delivery (0.3 per 1000). Of note, among those children born in the breech rather than in the cephalic presentation, these authors [24] found that a higher proportion of infants were born small for gestational age (SGA). However, these authors [23] did not distinguish foetal growth restriction among SGA neonates. In their interpretation, Bjellmo et al. [23] considered that “the overall higher risk for stillbirth and the higher proportion of infants born SGA among children born in the breech than in the cephalic presentation may suggest that foetuses with antenatal acquired risk factors for adverse outcomes are more likely to present in the breech than in the cephalic presentation at birth.” According to these authors, the findings were most likely explained by a combination of antenatal acquired risk factors for neonatal death with increased vulnerability to the birth process. Of note, in the TBT group, birth weights of less than 2.8 kg were also associated with adverse perinatal outcomes (*P* = 0.003) [10]. In fact, a limitation in the Norwegian study [24] was that, unlike Macharey et al., the authors did not distinguish foetal growth restriction among these SGA neonates. Indeed, in a large cohort study conducted with the National Health Service region in England through a multivariable analysis of 92,218 normally formed singletons delivered during 2009–2011 from 24 weeks of gestation, including 389 stillbirths, Gardosi et al. [25] showed that foetal growth restriction had the largest population attributable risk for stillbirth which was fivefold greater if it was not detected antenatally than when it was (32.0% v 6.2%). The above data suggest that some antenatal features associated with term breech presentation, notably foetal growth restriction, and some gestational disorders (such as uncontrolled gestational diabetes mellitus) could affect the prognosis in term breech cases. Previous data also support this conclusion; Luterkort M et al. [26] had previously reported in a prospective follow-up of 228 pregnancies with the foetus in the breech presentation in the 33rd gestational week that the 96 foetuses (42%) who remained in the breech presentation at delivery weighed 4.9% less than their vertex controls after adjustments were made for gestational age and had an increased frequency of oligohydramnios. Krebs et al. [27] later confirmed this association between breech presentation and foetal growth restriction from a register-based, case-control cohort of infants with cerebral palsy born between 1979 and 1986 in East Denmark.

In fact, as reported by Fox and Chapman [28], up to 21% of all foetuses adopt a noncephalic presentation at 28–29 weeks of gestation, and this proportion progressively decreases to 5% from 37 to 38 weeks [28]. Certain conditions, such as uterine malformation, can disturb both this continuous process of spontaneous cephalic version and normal foetal growth, thereby leading to increased term breech presentation rates in these cases [29]. This point highlights the importance of estimating foetal weight and well-being in cases of persistent breech presentation at term. Furthermore, even some cases of controlled GDM may be associated with excess foetal weight during the last weeks of pregnancy, leading to possible dystocia due to this overgrowth, or with other GDM-related complications, such as preeclampsia; thus, foetal weight estimates should be monitored closely beginning in the 37th week of gestation, with regular reassessment as long as the patient has not delivered.

#### The impact of strict criteria on the selection of vaginal delivery

From a broad perspective, in the most recent meta-analysis investigating the risks of planned vaginal breech delivery versus planned CS for term breech birth [21], the overall heterogeneity (I^2^ = 36%) was informative. The variability of neonatal mortality among 14 studies accounting for 74,094 breech vaginal deliveries was low (ranging from 0.08 to 0.37%). On the other hand, neonatal mortality was markedly higher in only 2 studies authored by Singh et al. [30] and Hannah et al. [2] (the TBT). These two studies [2, 30] accounted for 1099 breech vaginal deliveries (1.5% of births) and had perinatal mortality rates as high as 21 and 1.3%, respectively, for planned vaginal births (25.6% of perinatal deaths). The same was true for neurological morbidity, which was 3.4 and 1%, respectively, in the studies by Singh et al. [30] and TBT [2], while it ranged from 0.07 to 0.2% in the 14 other studies encompassing 74,094 breech vaginal deliveries conducted with the implementation of more stringent exclusion criteria for vaginal breech delivery.

In these 14 studies accounting for 74,094 breech vaginal deliveries, the retrospective observational cohort study from the Finnish Medical Birth Register [31] and the prospective observational study PREMODA [32] (as well as the more recent Norwegian Medical Birth Registry study) applied similar pre-established exclusion criteria for planned vaginal birth. In the PREMODA study, an increased absolute rate of perinatal death or serious neonatal morbidity was observed in both the planned vaginal group (1.60, 95% CI 1.14–2.17) and planned CS delivery group (1.45 [1.16–1.81]) with breech presentation among the total population of 264,105 births, but the planned vaginal group and the planned CS delivery group with breech presentation did not differ significantly for the combined outcome of foetal/neonatal mortality or serious morbidity (odds ratio [OR] = 1.10, 95% CI [0.75–1.61]). The Royal College of Obstetricians and Gynaecologists proposes comparable pre-established criteria for the management of term breech presentation, recommending that “women should be informed that a higher risk of planned vaginal breech birth is expected where there are independent indications for CS section and in circumstances such as a hyperextended neck on ultrasound, high estimated foetal weight (more than 3,800 g), low estimated weight (less than tenth centile), footling presentation, [and] evidence of antenatal foetal compromise” but considers that “the role of pelvimetry is unclear” [20]. Of note concerning this last point, Van Loon et al. showed in a randomized controlled trial [33] that the adequacy of pelvis size, as assessed by pelvimetry, improved the selection of delivery route. In line with them, two recent studies support this view [34, 35]. Other authors also included criteria for the adequate management and continuous monitoring of foetal heart rate during labour (which is common in maternity wards of most high-income countries but could be monitored intermittently in the TBT). Indeed, decreased variability and late decelerations are more prevalent during breech deliveries than vertex deliveries [36], and good labour progress is a predictor of better neonatal outcomes [37]. In the Finnish Medical Birth Register [31], 1270 women (43.6%) were selected as candidates for vaginal breech delivery, and the selection quality was confirmed by the low conversion rate of vaginal to CS breech delivery (11.4%). This rate was higher (36.1%) in the TBT [30].

As noted by methodologists [38], real-world prenatal patient care is subject to decision-making based on the continuous evaluation of risk factors, medical history, comorbidities, behavioural aspects, and other factors that indeed cannot be strictly reproduced in randomized controlled trials. For example, in the TBT [2], an upper limit of 4000–4500 g was given for estimated foetal weight. However, as the duration between randomization and delivery inevitably lengthened in the planned vaginal delivery group, a significantly higher number of macrosomic neonates were born in the planned vaginal delivery group (*P* = 0.002). In actuality, an informed woman who opts for vaginal delivery at 36 or 37 weeks of gestation usually changes her mind if she has not delivered several weeks later and if the clinician tells her that the birthweight will probably exceed 3800–4000 g, with an associated increased risk of adverse perinatal outcomes. Thus, in cases of even minor glycaemic disorder, special attention should be paid in the 37th week of gestation to foetal weight estimates and the possible occurrence of preeclampsia or associated gestational disorders; moreover, cases of SGA foetuses with possible foetal growth restriction should be closely followed, regardless of the delivery mode chosen by the patient [26, 39].

### How might we maximize patient benefit from a safe external cephalic version attempt?

With the restrictive practice of breech vaginal delivery in the last 15 years, national colleges of obstetricians (RCOG, ACOG, SOGC and RANZCOG) and FIGO updated their guidelines and recommended external cephalic version (ECV) at term to limit the increase in elective CS rate for cases of term breech presentation. However, recent data urge us to develop a broader perspective and an accurate assessment of the real impact of various ECV policies.

Indeed, the true impact of ECV may first be limited by the timely detection of breech presentation. In a retrospective cohort study of 394 consecutive cases of breech presentation at term, Hemelaar et al. [40] found that over two periods separated by 10 years (1998–1999 and 2008–2009), the proportion of breech presentations not diagnosed antenatally increased from 23.2 to 32.5% (*P* = 0.04), causing 52.8% of women who were eligible for ECV to miss an attempt in 2008–2009. The authors also reported that the proportion of women who declined ECV during the same period decreased significantly from 19.1 to 9.0%.

Eligibility is a second limitation. In Australia, a large-scale survey [41] showed that 22.3% of 32,321 singleton breech pregnancies were considered ineligible (due to oligohydramnios, antepartum haemorrhage or abruption, previous CS or pelvic abnormality, placenta previa, placenta accreta, or an infant with major congenital anomalies). In this survey [41], only 10.5% of the singleton breech pregnancies had an ECV. In a systematic review, Rosman et al. [42] identified 60 studies that reported 39 different contraindications and five guidelines with 18 contraindications (varying from five to 13 contraindications per guideline), with oligohydramnios being the only contraindication that was consistently mentioned in all guidelines. Thus, there was no general consensus on the eligibility of patients for ECV, but contraindications generally include all conditions in which this procedure may be associated with a particular risk for the foetus or mother. These conditions include the following: severe intrauterine growth restriction, abnormal umbilical artery Doppler index and/or nonreassuring foetal heart rate, which may require an emergency CS birth; foetuses with a hyperextended head and significant foetal or uterine malformations, which may carry a particular foetal risk; rhesus alloimmunization, which might be reactivated by the procedure; and recent vaginal bleeding or ruptured membranes, which were associated with cord prolapse in 33% of reported cases after ECV attempt [43].

If CS or rapid delivery is indicated for another obstetric condition, ECV is also contraindicated, notably in cases of placenta previa, severe preeclampsia, and increased risk of placental abruption. Other situations, such as maternal obesity, nonsevere SGA foetuses, and nonsevere oligohydramnios, merely decrease the likelihood of ECV success. In contexts such as severe oligohydramnios or multiple gestations, ECV is simply impracticable, except for a second twin after delivery of the first. Furthermore, previous uterine surgery (CS delivery, myomectomy, or hysteroplasty) is considered a relative contraindication for ECV by some but not all authors [44]. On the other hand, in patients with gestational diabetes mellitus, incomplete or uncontrolled glucose levels are associated with an increased risk of foetal macrosomia in late pregnancy, and even if the estimated foetal weight seems compatible with a planned vaginal delivery when the mode of delivery is discussed, rapid foetal growth during the last weeks may lead to major difficulties during delivery. Therefore, in such a context, we believe there is potential for a particular benefit from successful ECV at 36 weeks.

### Predictors of successful ECV

Pinard previously observed that unengaged breech presentation is an important predictor of successful ECV [45]; the same observation was made by Lau et al. [46], Aisenbrey et al. [47], and Hutton et al. [48]. In the large series of 1776 ECVs published by Hutton et al. [48], descent and impaction of the breech foetus were the most discriminating factors for predicting successful ECV, regardless of parity. Other predictors of success include parity [45, 47, 49, 50], abundant amniotic fluid [49–51], nonfrank breech presentation [47], gestational age under 38 weeks [43], and posterior placenta [50]. In contrast, nulliparity and tense uterus are associated with a lower likelihood of success [44, 48, 52].

Velzel et al. [53] recently reviewed prediction models, most of which were developed without any external validation, and found that the most reliable predictors of successful ECV were nonimpacted breech presentation, parity and uterine softness (which usually go hand in hand), normal amniotic fluid index, posterior placental location, and, as noted by Pinard [45], foetal head in a palpable situation. These criteria might be used to support patient counselling and decision-making about ECV and to reduce the proportion of women declining ECV, particularly in the most favourable situations for ECV.

### Obstetric outcomes after an ECV attempt

De Hundt et al. [54] conducted a systematic review and meta-analysis and showed that women who have had a successful ECV for breech presentation are at increased risk for CS delivery (OR 2.2; 95% CI 1.6–3.0) and instrumental vaginal delivery (OR 1.4; 95% CI 1.1–1.7) compared with women with spontaneous cephalic presentation. Interestingly, stratification by time delay between successful ECV and delivery revealed a trend for increased risk of CS during the first week after ECV [55]. Furthermore, in a cohort of 301 women with successful ECV, De Hundt et al. [56] found that nulliparity was the only of seven factors that predicted the risk of CS and instrumental vaginal delivery (OR 2.7; 95% CI 1.2–6.1). Based on a retrospective, population-based cohort study using the CDC’s birth data files from the US in 2006, Balayla et al. [57] also showed that relative to breech controls without an ECV attempt, cases of ECV failure with persistent breech presentation and labour attempts were associated with increased odds of CS delivery (adjusted OR 1.38; 95% CI 1.21–1.57), assisted ventilation at birth (aOR 1.50; 95% CI 1.27–1.78), 5-min Apgar score < 7 (aOR 1.35; 95% CI 1.20–1.51), and neonatal intensive care unit admission (aOR 1.48; 95% CI 1.20–1.82).

This information should also be considered in the dialog with women regarding the way in which late pregnancy and delivery should be managed based on existing data, their own situations and their wishes.

The true benefit of an active and systematic ECV policy is widely appreciated [58, 59], and such evaluation may be subject to bias. Burgos et al. [58] found that their policy decreased the rate of breech presentation at delivery by 39.0% and decreased the CS rate for cases of breech presentation at term from 59 to 44%. On the other hand, Coppola et al. [59] reported that their CS rate was not significantly reduced in the planned ECV group, even after adjustments were made for age, parity and previous CS delivery. Thus, each perinatal centre should implement an appropriate and coherent policy in accordance with the prevalence of pathologies in the population.

### Towards a consensus for a global shared vision and management of term breech presentation that could include the following

- A policy of breech presentation screening at 36 weeks of gestation is efficient and cost effective [60].
- Such screening should allow timely ECV and a careful evaluation of potential underlying antenatal risks, considering obstetric history, estimated foetal weight/growth and potential gestational disorders [23–27, 29].
- Foetal weight estimates based on clinical and ultrasound examinations are essential, despite the large confidence interval of all available algorithms for producing such estimates. Vaginal birth may be excluded when the estimated foetal weight approximates the upper limit used for selection in most national guidelines (3800 g) [18–20], particularly in the absence of previous successful vaginal delivery.
- Before vaginal delivery is considered, clinical pelvic examination is universally recommended to rule out pathological pelvic contraction. Radiologic or magnetic resonance imaging (MRI) pelvimetry is not universally conducted [20, 23, 24, 31, 32]. However, Van Loon et al. [33] demonstrated in a randomized controlled trial that the use of MRI pelvimetry in breech presentation at term allowed better selection of delivery route, with a significantly lower emergency CS rate. More specifically, several recent studies [34, 35] have evaluated the contribution of pelvimetry and found that MRI pelvimetry provided useful criteria for the preselection and counselling of women with breech presentation and the desire for vaginal delivery. Therefore, pelvimetry is diversely used in Europe for the preselection and counselling of women (particularly nulliparous women) with breech presentation and is specifically used in regions where vaginal delivery is still considered an option [35].
- In cases of failed ECV with persistent breech presentation, this policy should allow customized care tailored to each situation in the last weeks of pregnancy.
- A discussion with the informed patient is essential. One must thoroughly consider the experience of the health care team/the availability of clinical skills required for conducting a vaginal breech delivery and carefully select women who are eligible for planned vaginal delivery (considering obstetric history and the criteria described above for the choice between planned vaginal and CS deliveries) [20, 23, 24, 26, 28].
- Regardless of the planned mode of delivery [22], adequate follow-up during the last weeks of pregnancy is mandatory, with particular consideration of possible associated underlying disorders (particularly foetal growth restriction or excessive foetal weight in cases of gestational diabetes mellitus) [24–26]. Thus, the foetal weight estimation should be carefully considered in the 37th week of gestation, even in cases of minor glycaemic disorder, with regular reassessments and a plan for CS delivery if the patient remains pregnant for many more weeks and if foetal weight estimates reach approximately 3600–3800 g.
- If vaginal delivery is planned, careful labour management by a skilled team is needed, accompanied by continuous foetal heart rate monitoring [36] and a particular focus on the rate of progress in the second delivery stage [37]. When such conditions are not or cannot be fulfilled, a planned CS may be the best choice.
- When a CS has been planned, adequate follow-up during the last weeks of pregnancy and careful calculation of the delivery date are needed, taking into account possible comorbidities and gestational disorders.

## Conclusion

Term breech presentation is a condition for which personalized obstetrical care is particularly needed. The best way is likely to be as follows: first, efficiently screen for breech presentation at 36–37 weeks of gestation; second, thoroughly evaluate the maternal/foetal condition, foetal weight and growth potential, and the type (frank, complete, or footling) and mobility of breech presentation; and three, consider the obstetric history and pelvic size/conformation. The management plan, including ECV and follow-up during the last weeks, should then be organized taking into account antenatal risk factors on a case-by-case basis by a skilled team after informing the woman, discussing her personal situation and criteria and helping her make a rational decision. Foetal overgrowth or growth restriction and/or oligohydramnios may necessitate timely CS, and the mode of delivery should be re-evaluated as necessary according to obstetric conditions (e.g., estimated foetal weight and Bishop score).

## Data Availability

Not applicable.

## Abbreviations

ACOG: American College of Obstetricians and Gynecologists
CS: Caesarean section
ECV: External cephalic version
FIGO: International Federation of Gynecology and Obstetrics
RANZCOG: Royal Australian and New Zealand College of Obstetricians and Gynaecologists
RCOG: Royal College of Obstetricians and Gynaecologists
SMM: Severe maternal morbidity
SOGC: Society of Obstetricians and Gynaecologists of Canada
TBT: Term breech trial
